# Bacterial and Fungal Co-Occurrence in the Nudibranch, *Pteraeolidia semperi*

**DOI:** 10.3390/life12121988

**Published:** 2022-11-28

**Authors:** Ming Sheng Ng, Nathaniel Soon, Ying Chang, Benjamin J. Wainwright

**Affiliations:** 1 Yale-NUS College, National University of Singapore, 16 College Avenue West, Singapore 138527, Singapore; 2 Department of Biological Sciences, National University of Singapore, 16 Science Drive 4, Singapore 117558, Singapore

**Keywords:** bacteria, fungi, co-occurrence, marine microbiome, core microbiome

## Abstract

Despite the increasing recognition and importance surrounding bacterial and fungal interactions, and their critical contributions to ecosystem functioning and host fitness, studies examining their co-occurrence remain in their infancy. Similarly, studies have yet to characterise the bacterial and fungal communities associated with nudibranchs or their core microbial members. Doing this can advance our understanding of how the microbiome helps a host adapt and persist in its environment. In this study, we characterised the bacterial and fungal communities associated with 46 *Pteraeolidia semperi* nudibranch individuals collected from four offshore islands in Singapore. We found no distinct spatial structuring of microbial community, richness, or diversity across sampling locations. The bacterial genera *Mycoplasma* and *Endozoicomonas* were found across all samples and islands. The fungal genus *Leucoagaricus* was found with the highest occurrence, but was not found everywhere, and this is the first record of its reported presence in marine environments. The co-occurrence network suggests that bacterial and fungal interactions are limited, but we identified the bacterial family *Colwelliaceae* as a potential keystone taxon with its disproportionately high number of edges. Furthermore, *Colwelliaceae* clusters together with other bacterial families such as *Pseudoalteromonadaceae* and *Alteromonadaceae*, all of which have possible roles in the digestion of food.

## 1. Introduction

Microbes (bacteria, fungi, archaea, and viruses) are fundamental to life where they are found in nearly every habitat and organism on the planet [1]. They have critical roles in the mediation of ecosystem functioning in terrestrial and marine ecosystems and have key roles in biogeochemical and nutrient cycling [2]. Similarly, microbes can form symbiotic relationships with their hosts that can promote health, fitness, and aid in metabolism [3].

Advances in DNA sequencing technology and high-performance computing have rapidly accelerated our understanding of microbial diversity in environmental and host-associated systems in a variety of locations and species [4,5]. However, in comparison, studies examining the co-occurrence of bacteria and fungi within their host, particularly for marine hosts, are in their infancy despite their recognised importance and their likely synergies. Microbial ecosystems are largely dominated by bacteria and fungi, and unravelling their interactions can allow a better understanding of evolutionary relationships and the maintenance of host health, among other processes [6]. These interactions can be revealed by network analysis of co-occurrence, which also identifies potential keystone species or ecological core members [7] that have disproportionate influences in microbial communities in comparison to others [8]. In this study, we examine the bacterial and fungal communities of a widespread nudibranch from the waters of Singapore and look for evidence of co-occurrence.

Nudibranchs (order: Nudibranchia) are a group of charismatic soft-bodied marine gastropod molluscs that are often noted for their spectacular colouration and striking forms. These marine invertebrates generally have short lifespans of four to five months [9,10] and have evolved to lose their shells as they mature [9]. The majority are adapted to disperse only locally, frequently emerging as fully formed, albeit small individuals [10]. This limited dispersal potential leads to high levels of genetic differentiation between populations separated by as little as one hundred meters [11]. This low dispersal potential facilitates evolution and high nudibranch diversity, especially in the shallow waters of tropical archipelagos [12,13] where molecular work has revealed a high degree of cryptic speciation [14,15,16,17] with new species frequently being described [18].

Nudibranchs are frequently studied for their ability to produce [17] and sequester secondary metabolites from their food sources such as hydroids, algae, tunicates, and corals [18]. These secondary metabolites play crucial roles in promoting host fitness, especially by defending against predation in the absence of a protective shell, where these metabolites can make the host unpalatable or toxic to potential predators [18]. More recently, the role of the microbial community in the production of chemical compounds via biosynthesis was also considered [19], with nudibranch-associated bacteria found to produce biosynthetic products that display antimicrobial and antitumor activities [19,20,21,22]. The nudibranch *Rostanga alisae* was also discovered to be symbiotic with the bacterial genus *Synechoccus,* which likely has crucial roles such as photosynthesis and the production of defensive toxins [23]. Symbiotic bacteria can also supplement fatty acid for their nudibranch hosts [24] or have important nutritional roles by aiding in the digestion of sponge chitin and sponging [23]. Besides bacteria, numerous fungal species have been isolated from nudibranchs, with many of the isolates inhibiting the growth of significant pathogenic bacteria such as *Vibrio harveyi* and *Vibrio vulnificus* [25,26,27].

Despite the importance of fungal and bacterial associates in the host microbiome, studies that investigate both the core microbiome and mycobiome remain greatly understudied, especially in the lesser-studied nudibranchs. Furthermore, no prior study has investigated the interactions between bacteria and fungi associated with nudibranch hosts. A core microbiome or mycobiome is defined as the group of bacteria or fungal taxa characteristic of a specific host [28]. Identifying these ecological and common core, or shared, taxa [7], can advance our understanding of how the structure and composition of the microbiome adapt to the host’s ecology and habitat, allowing deeper insight into host biology and any symbiotic relationships that are present.

In this study, we used DNA metabarcoding techniques to examine the bacterial and fungal communities associated with the nudibranch, *Pteraeolidia semperi*, commonly known as the ‘blue dragon’ nudibranch. This species is widespread throughout its native Indo-Pacific range. They are one of the most common species found in Singapore at water depths of less than three meters and throughout the intertidal zone. Therefore, we performed spatial analyses and describe the bacterial and fungal communities associated with 46 individuals collected from four islands in the waters of Singapore. We investigated the common core microbial members associated with the nudibranch, *Pteraeolidia semperi,* and identified potential keystone microbial taxa through the co-occurrence network. On account of their limited dispersal, we hypothesise that microbial community structure will differ across sites, with individuals collected from the same islands showing a more similar microbial community in comparison to those collected from other islands.

## 2. Materials and Methods

**Figure 1:**
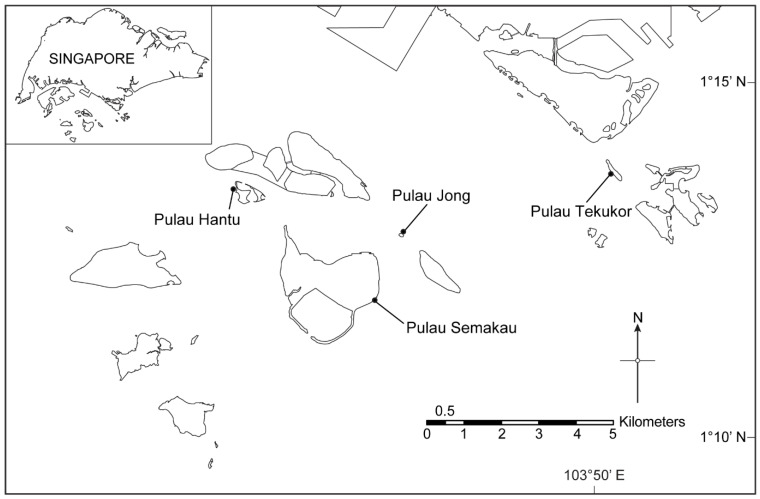
Sampling sites from the islands south of Singapore.

A total of 46 *Pteraeolidia semperi* [29] specimens were collected between November 2020 and April 2021 from the reefs surrounding four different islands south of mainland Singapore: Pulau Hantu, Pulau Jong, Pulau Semakau, and Pulau Tekukor (Figure 1). Of those, 26 individuals were from Pulau Hantu, 5 from Pulau Jong, 5 from Pulau Semakau, and 10 from Pulau Tekukor. These islands were selected as *Pteraeolidia semperi* is known to be found around them. In brief, the reefs of the four islands are dominated by dead corals and sediment with a similar coral cover of 20–30% [30,31,32], except for Pulau Jong with a lower coral cover of only 5% [31]. Subtidal sampling was conducted via SCUBA, and specimens were identified based on known anatomical descriptions [33]. Samples were kept in 100% ethanol prior to DNA extraction, and species identification was confirmed via an examination of the radula, partial cytochrome c oxidase subunit I (COI) sequencing, and phylogenetic analysis as described in Soon et al. (in review) [34]. Briefly, COI sequences were aligned, a maximum likelihood tree was then constructed and Bayesian inference of phylogeny was then performed to confirm species identification. Only species confirmed as *Pteraeolidia semperi* were used in the analysis.

### 2.1. DNA Extraction and Amplification

DNA was extracted from the entire individual with a Qiagen DNeasy^®^ Blood and Tissue Kit following the manufacturer’s protocol. Amplification of the V4 region of the 16S SSU rRNA was performed with the 515F and 806R primer pair, modified to include Illumina adapters and a unique barcode [35]. PCR cycling conditions followed that of Oh et al. [36]: each reaction was performed in a total volume of 25 µL, containing 12.5 μL KAPA PCR Buffer, 0.1 μL of KAPA 3G Enzyme (Kapa Biosystems, Inc., Wilmington, MA, USA), 0.75 μL of each primer at 10 μM, 1 μL of undiluted template and nuclease-free water to 25 μL. PCR cycling conditions began with an initial denaturation step at 94 °C for 3 min, followed by 35 cycles of 94 °C for 45 s, 50 °C for 60 s, and 72 °C for 90 s, with a final extension at 72 °C for 10 min.

Amplification of the fungal ITS region was performed under the same conditions used in Lee et al. [5]: we amplified the internal transcribed spacer 1 (ITS1) region of fungal DNA using the ITS1F and ITS2 [37] primer pair, modified to include a unique barcode and Illumina adapters [38]. PCR cycling conditions began with an initial denaturation step at 95 °C for 3 min, followed by 35 cycles at 95 °C for 20 s, 53 °C for 15 s, and 72 °C for 20 s, with a final elongation at 72 °C for 1 min. Negative extraction and PCR controls were included and sequenced to identify any potential contamination issues.

All PCR products were then visualised on a 1% TAE buffer agarose gel to confirm amplification, then cleaned and normalised using SequalPrep™ Normalization Plates (Invitrogen, Frederick, MD, United States). Fungal and bacterial samples were sequenced independently on the Illumina MiSeq platform (600 cycles, V3 chemistry, 300 bp paired-end reads), both with a 30% PhiX spike by Macrogen, Inc. Both libraries were sequenced with other unrelated projects.

### 2.2. Bioinformatics

The bioinformatics workflow and analyses were conducted on the R platform [39]. Bioinformatic workflows, comprising quality filtering and taxonomic assignment, were adapted from the DADA2 [40] ITS Pipeline Workflow V1.81 (https://benjjneb.github.io/dada2/ITS_workflow.html, accessed on 15 June 2022) for fungal sequences, with fungal reads truncated at 300 bp and 200 bp for forward and reverse reads, respectively. The DADA2 Pipeline Tutorial V1.16 (https://benjjneb.github.io/dada2/tutorial.html, accessed on 20 June 2022) was used for bacterial sequences and reads were truncated at 220 bp and 150 bp for forward and reverse reads, respectively. Additionally, the R package *decontam* (v1.16.0) [41] was used to identify and remove any contaminant DNA sequences via the prevalence-based identification method.

Amplicon sequence variants (ASVs) not assigned to fungi or bacteria in their respective datasets were removed before downstream analysis. Raw sequence counts were converted to relative abundance data for use in all subsequent analyses. Unless otherwise indicated, all analyses were performed on the bacterial and fungal data independently. Rarefaction curves were first produced with the *rarecurve* function from the *vegan* package (v2.6.2) [42] to ensure sufficient sampling depth and full recovery of diversity for each sample. The Shannon diversity index (H′) and species richness were calculated for each sample via the *vegan* package [42]. Analysis of variance (ANOVA) was performed to investigate whether fungal or bacterial communities had significantly different diversity or species richness across sites. All models were checked for normality and homoscedasticity via diagnostic plots to ensure model validity.

To visualise microbial communities associated with *P. semperi* from each of the four sample sites, principal coordinate analysis (PCoA) plots were constructed for both bacterial and fungal communities separately with both weighted UniFrac distances (to account for phylogenetic dissimilarity [43]) and Bray–Curtis dissimilarity matrix via the *phyloseq* package (v1.40.0) [44]. Permutational analysis of variance (PERMANOVA) was then conducted via the *adonis2* function from the *vegan* package [42] to investigate if the bacterial and fungal community compositions differed significantly across nudibranchs from different sites. Pairwise comparisons were conducted using the *emmeans* package [45] for ANOVA models and *pairwiseAdonis* package [46] for PERMANOVA models. The assumption of homogenous group dispersion was first assessed with *betadisper* [42] before conducting PERMANOVA.

The common and ecological core of fungal and bacterial associates of the nudibranch *P. semperi* were identified with the occurrence method and through network analysis, respectively, as reviewed in [7]. The occurrence method was assessed with the *core_members* function from the *microbiome* package [47], which identifies the most widespread taxa found across the samples. Network analysis was conducted with the *SpiecEasi* package [48] and subsequently visualised with the Fruchterman–Reingold layout algorithm in Gephi [49] following [50]. In general, the *SpiecEasi* (SParse InversE Covariance Estimation for Ecological Association Inference) method allows the inference of correlations between populations while addressing the lack of independence between microbial compositions and spurious links from traditional correlation analyses. Keystone members were then identified with the mean degree, or the number of edges a node has [51].

All samples were collected under permit number NP/RP20-088b, issued by the National Parks Board of Singapore.

## 3. Results

A total of 9,474,471 and 488,692 reads were generated on the Illumina MiSeq platform for bacterial and fungal data, respectively. After removing chimeric sequences and low-quality reads, 5,972,183 bacterial and 276,984 fungal sequences were retained for analysis (Table S1). All samples were used in the bacterial analysis. Due to low sequencing depth, three samples (HG06, HN11 & TK08) were discarded in the fungal analysis. Rarefaction curves show that sufficient depth was attained to reach asymptote, indicating all fungal and bacterial diversity (with the exception of some fungal reads which did not plateau) was recovered for samples to use in downstream analyses (Figure S1).

**Figure 2:**
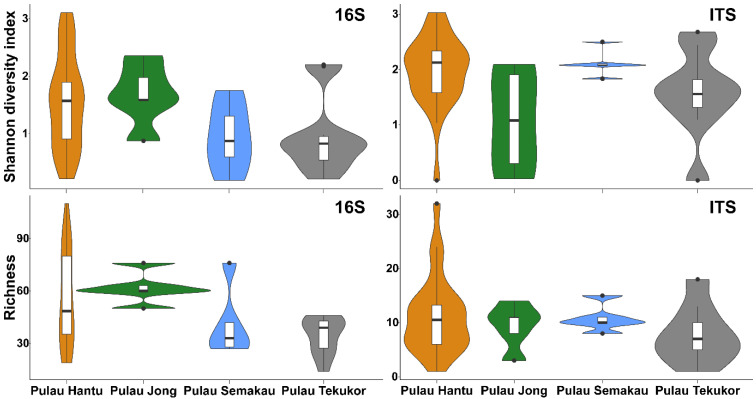
Violin and boxplots of Shannon diversity index and richness of bacteria (16S) and fungal (ITS) communities of nudibranchs sampled from the four sites. Bold horizontal bars indicate the means while the points indicate outliers.

Bacterial communities associated with *P. semperi* had similar Shannon diversity (*p* > 0.05, Figure 2) but significantly different species richness across sites (*p* < 0.05, Table S2). However, the pairwise analysis revealed no site pairs with nudibranch-associated bacterial communities of significantly different species richness. Conversely, while the fungal communities had similar species richness (*p* > 0.05), they had significantly different Shannon diversity across the nudibranchs from the four sites (*p* < 0.05, Table S2). Likewise, the pairwise analysis showed no island pairs with nudibranch-associated fungal communities with significantly different Shannon diversity indexes.

**Figure 3:**
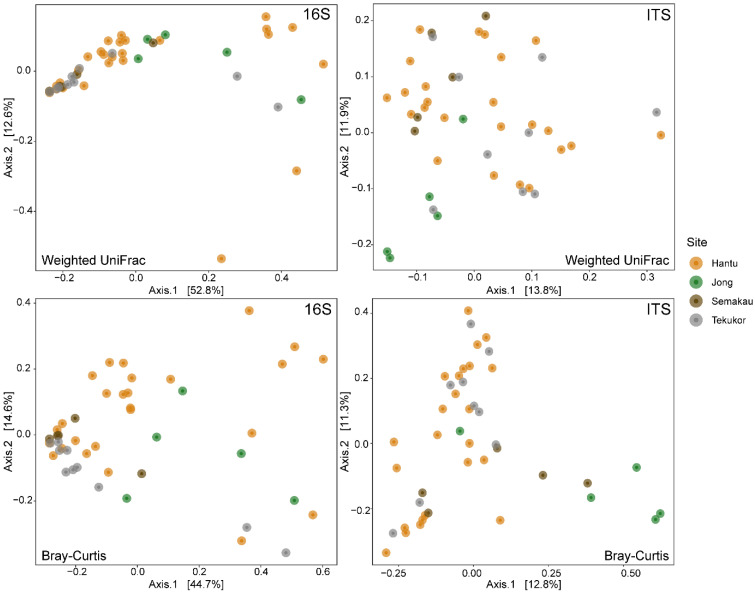
Principal coordinate analysis (PCoA) with weighted UniFrac distances (**top row**) and Bray–Curtis (**bottom row**) on bacterial (**left**) and fungal (**right**) communities of nudibranchs sampled across the four sites.

Principal coordinate analysis (PCoA) plots with weighted UniFrac and Bray–Curtis distances showed that bacterial communities did not exhibit distinct clustering across the four sites (Figure 3). This is supported by the PERMANOVA results with both weighted UniFrac and Bray–Curtis distances, as each indicates significant differences in bacterial communities across sites (*p* < 0.05, Table S3), but the post-hoc multi-level pairwise comparisons show that no site pairs were significantly different (Table S4). Bacterial communities did not have significantly different dispersions across nudibranchs sampled from the four different sites.

PERMANOVA performed on fungal data indicated significantly different communities across sites with both distance metrics (*p* = 0.001, Table S3). However, the PCoA plots showed no distinct site-specific clustering of the fungal communities (Figure 3). As such, the PERMANOVA results are likely affected by the non-homogenous dispersion of data as indicated by the *betadisper* results with both distance metrics (Weighted UniFrac: F = 6.29, *p =* 0.001; Bray–Curtis: F = 6.038, *p* = 0.001).

**Figure 4:**
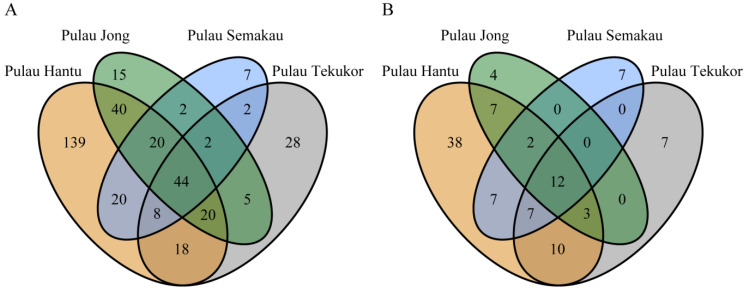
Number of (**A**) bacterial and (**B**) fungal ASVs shared between nudibranchs from the four different islands.

All four sites had bacterial and fungal ASVs unique to them (Figure 4). Pulau Hantu had the highest number of unique ASVs with 139 bacterial ASVs and 38 fungal ASVs, while Pulau Semakau had the lowest number of unique ASVs with 7 bacterial ASVs and 7 fungal ASVs. However, these were rare ASVs that had very low relative abundance. For instance, despite having 139 bacterial ASVs unique to Pulau Hantu, they only comprised 1.02% ± 0.31% (mean ± SE) of the entire bacterial community across the 26 nudibranch samples from Pulau Hantu. On the other hand, the shared bacterial and fungal ASVs across the four sites made up the bulk of the nudibranch’s microbiome. The 44 bacterial ASVs shared among all four sites comprised on average 92.15% ± 1.44% of the bacterial community across the 46 nudibranch samples. Likewise, the 12 fungal ASVs shared across the four sites comprised on average 45.00% ± 3.78% of the fungal community across the 43 samples. The full list of ASV identities unique to each site and shared across all four sites is listed in the supplementary information (Table S5).

**Figure 5:**
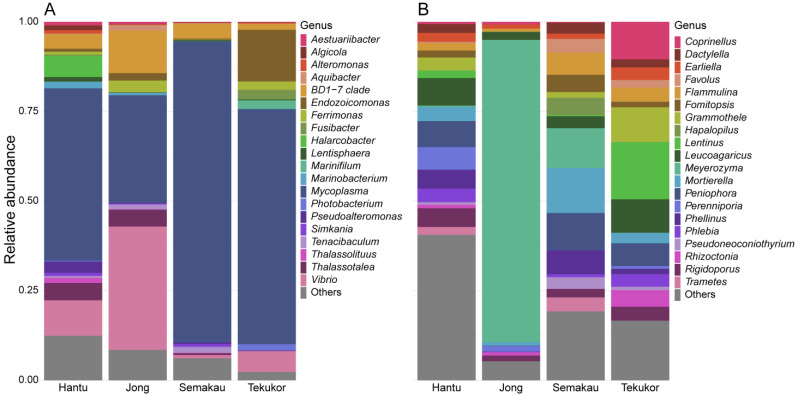
Stacked bar plot of the top 20 (**A**) bacterial and (**B**) fungal genera for each island sampled. See Figures S2–S5 for additional plots at other taxonomic ranks.

Of the 44 shared bacterial ASVs found across all four sites, only the genera Mycoplasma and Endozoicomonas were found in all 46 samples, while Vibrio was found in 45 of 46 samples, missing in a sample collected from Pulau Semakau (Figure 5A). The bacterial genus clade BD1-7 was present in 42 of the 46 samples, while absent in three samples from Pulau Hantu and in one sample from Pulau Semakau. The top five bacterial genera with the highest relative abundances (occurrence) across the nudibranch samples were Mycoplasma (56.55% ± 4.49%), Vibrio (8.50% ± 2.07%), clade BD1-7 (5.32% ± 1.70%), Endozoicomonas (3.76% ± 1.20%), and Thalassotalea (3.17% ± 0.68%).

Conversely, no fungal genera were found across all samples. The fungal genus Leucoagaricus had the highest prevalence where it was recovered from 29 of the 43 samples, followed by Mortierella and Flammulina which were both found in 19 samples (Figure 5B). The top five fungal genera with the highest relative abundances (occurrence) were Meyerozyma (8.99% ± 3.65%), Peniophora (8.59% ± 2.71%), Leucoagaricus (8.51% ± 1.61%), Rigidoporus (4.26% ± 1.11%), and Mortierella (3.74% ± 0.85%). No distinct patterns of prevalence or occurrence were observed in the samples collected across the four sites for either bacterial or fungal communities.

**Figure 6:**
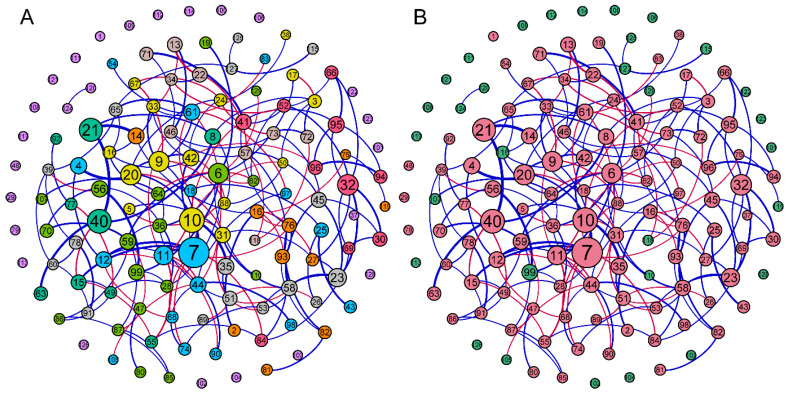
Network connection of bacterial and fungi families associated with nudibranchs, with the colours representing different modules in (**A**) and kingdoms in (**B**). Node size scaled to betweenness centrality, edge thickness scaled to strength of correlation, and edge colour represents positive (blue) or negative (red) correlation. The numbers on the nodes refer to the identity of the ASVs.

The network analysis clustered only 13 of the 31 fungal families within the network with 93 of the 98 bacterial families (Figure 6). The average network distance between all pairs of nodes (average path length) was 4.28 edges (connections) with a diameter of 10 edges. The clustering coefficient (the degree to which they tend to cluster together) was 0.19 with a modularity index of 0.71 (values > 0.4 suggest that the network has a modular structure). Overall, the network was moderately well-connected with an average of 2.82 edges. Of the fungal families, Hymenochaetales (Incertae sedis) (node 116) had the highest number of edges at four, followed by Marasmiaceae (node 127) and Debaryomycetaceae (node 99) with three edges each. The bacterial family Colwelliaceae (node 7) had the greatest number of edges at 11, followed by Crocinitomicaceae (node 6) at nine edges. All data and centrality metrics can be found in the supplementary information (Table S6).

## 4. Discussion

In this study, we characterised the bacterial and fungal communities associated with the nudibranch *Pteraeolidia semperi* collected from four different islands in the waters of Singapore. We show that the microbial diversities and community structures are generally similar across all four examined sites with a lack of clustering seen in the principal coordinate analysis plots. Through the occurrence method, we found that the bacterial genera *Mycoplasma* and *Endozoicomonas* were detected across all samples, and *Vibrio* was only absent in one sample from Pulau Semakau. In the fungal dataset, the genus *Leucoagaricus* had the highest occurrence where it was found in 29 of the 43 samples. There were no distinct occurrence patterns for either fungal or bacterial genera across the four sampled sites. Potential keystone taxa in the nudibranch’s microbial community were identified through the network analysis of the co-occurrence of both bacterial and fungal families. The bacterial family *Colwelliceae* and the fungal taxon *Hymenochaetales* had the highest number of edges among their respective domains, although cross-domain interactions were limited.

There were unique bacterial and fungal genera specific to each site, although most of them were found at very low relative abundances. For instance, *Psychrobium* was the most abundant bacterial genus only found on nudibranch samples from Pulau Hantu with an average relative abundance of 0.33%. These are chemo-organotrophic and psychrophilic bacteria that thrive on dissolved organic material [52] and were previously found living within mussels [53]. Conversely, the most abundant fungal genus unique to Pulau Hantu was *Sebipora* with an average relative abundance of 3.49%, but made up 45.30% of the fungal community from one sample. Fungi from this genus are known to cause white-rot diseases in trees [54,55]. They have not been previously reported in marine environments and their roles and functions in *P. semperi* remain to be explored. Nevertheless, the bulk of the bacterial and fungal communities consisted of the 44 bacterial and 12 fungal genera shared among all four sites, with an average of 92.15% and 45.00% of the bacterial and fungal communities, respectively, across all nudibranch samples. The identification of these shared and unique bacterial and fungal genera can provide better direction towards understanding the roles and functions of nudibranch-associated microbial members, especially the shared bacterial and fungal genera across the four sites.

This large proportion of shared bacterial and fungal genera across the four sites can help explain the lack of site-specific effects on the composition of fungal and bacterial communities of *P. semperi*. While the fungal communities associated with *P. semperi* exhibited small but significant differences in their composition across the four sites, the lack of distinct clustering in the principal coordinate analysis suggests that these changes were likely driven by the heterogeneous dispersion of data. Likewise, bacterial communities were not significantly different across sites. This is in agreement with previous work examining fungal communities associated with *Pocillopora acuta* corals from similar sampling sites in Singapore [56], where the lack of difference is likely due to the generally well-mixed waters [57] and the small spatial scale. However, previous metabarcoding efforts of different marine hosts in Singapore including seagrasses [58], mangrove trees [5], and reef macroalga [36] all show significant differences in microbial communities across different sampling locations. Despite so, together with the similar Shannon diversity indexes and species richness, our work indicates that microbial diversity and composition associated with *P. semperi* are similar across the four sites sampled in Singapore. This is likely due to a combination of the small geographic distance between sample sites and the correspondingly high connectivity between them, and a shared microbial core ubiquitous across the four sites essential to the functioning and survival of the nudibranch *P. semperi*.

Three notable bacterial genera, *Mycoplasma, Endozoicomonas,* and *Vibrio*, were found across all samples (except one for *Vibrio*). These genera are commonly associated with the gut microbiome of marine invertebrate and vertebrate hosts [59,60,61,62], including other species of nudibranchs [20]. From the class Mollicutes, *Mycoplasma* bacteria are generally considered to be parasitic organisms that rely on their hosts completely for nutrients [63,64]. *Mycoplasma* frequently forms biofilms that are resistant to stress such as heat and desiccation which contributes to their persistence in marine environments and on hosts [65]. The roles and functions of both *Vibrio* and *Endozoicomonas* are highly variable, ranging from nutrient cycling to pathogenic [61,66,67]. Interestingly, *Endozoicomonas* bacteria are shown to correlate with the presence of photosymbionts in many marine invertebrates, including corals [68,69], giant clams [70], and sponges [71], suggesting a degree of symbiosis between them. The nudibranch, *P. semperi,* is known to harbour photosynthetic Symbiodiniaceae [72], which could explain the ubiquity of *Endozoicomonas* bacteria found here. Additional work is needed to confirm whether this is a symbiotic relationship, although the co-occurrence in more than one taxon is suggestive of such. However, it is important to note that the near ubiquity of these three bacteria genera found to associate with nudibranchs could simply reflect their prevalence in the environment instead of playing core roles in promoting the fitness of *P. semperi.*

There was no fungal genus found across all samples. The genus *Leucoagaricus* had the highest occurrence (29 of 43 samples) followed by *Mortierella* and *Flammulina* (both 19 of 43 samples). Despite having the highest occurrence, there are no previous reports of *Leucoagaricus* in strictly marine environments; however, four species within this genus have been found in sandy coastal habitats [73,74,75]. Comprising about 90 species, *Leucoagaricus* are mostly found on forest soil [76,77], with the most notable *L. gongylophorous* known for being farmed by its symbiotic partner, the fungus-growing ants, to be later consumed [78]. Several members of *Leucoagaricus* can also break down leaf matter through the production of enzymes such as lignocellulase [79], cellulase, and xylanase [80]. As *P. semperi* are often found on algae-covered surfaces [81], and this algae likely forms part of their diet [82], it is possible that *Leucoagaricus* may play a role in degrading algal cellulose and xylan. *Flammulina* has previously been detected in marine hosts such as corals and coralline algae [83], and has a wide distribution in temperate regions [84]. The fungal genus *Mortierella* is also commonly found on terrestrial soils [69], although it has previously been found in extreme marine environments such as Antarctica [85] and deep-sea sediments [86].

The microbial co-occurrence network highlighted several taxa with a high number of edges that may serve as keystone taxa. The edges in a co-occurrence network refer to the connections one taxon has to another, and taxa with a high number of edges are often regarded as keystone [50] as their removal can cause a drastic shift in the composition and functioning of a microbiome [87]. Overall, only a few fungal taxa were connected in the network, with most only having one edge indicating limited cross-domain interactions between bacteria and fungi. Of the 13 fungal families within the network, the taxon *Hymenochaetales* had the greatest number of edges at four. These fungi are commonly known as white rot fungi and are usually associated with diseases in terrestrial plants [88] containing ligninolytic enzymes [89], although they have also been found to associate with marine organisms such as nematodes [90] and corals [91]. *Hymenochaetales* is most positively correlated with *Kangiellaceae* and *Oleiphilaceae,* both common marine bacteria with roles in nutrient cycling. *Hymenochaetales* could play a role in digesting lignin found in algae [92] to amino compounds and hydrocarbons, which *Kangiellaceae* [93] and *Oleiphilaceae* [94] can then utilise.

The bacteria family *Colwelliaceae* had a disproportionately central role in the network with 11 edges. *Colwelliaceae* bacteria are strictly marine in distribution with many able to hydrolyse starch [95], which could aid *P. semperi* in degrading algal food sources [96]. Many members from this family are also known to digest chitin [97] found in hydroids [98], which is a major food source for the nudibranchs [99]. In fact, within the same module (group of tightly interconnected nodes), *Colwelliaceae* is most positively connected to *Pseudoalteromonadaceae* [100] and *Alteromonadaceae* [101], both are known to degrade agar and starch, also major components of algae. As such, members within this module are likely to interact and co-exist to synergistically aid *P. semperi* in the digestion of algae. Similarly, previous studies on the nudibranch, *Rostanga alisae,* also associated bacteria capable of hydrolysing major components of its sponge diet; this ability also likely contributes to host feeding success [23]. It is also important to note that the bacterial genera *Pseudoalteromonas* from the family *Pseudoaltermonadaceae* were found to have anti-microbial abilities in nudibranchs that may help them prevent diseases [22,102].

In conclusion, our study used metabarcoding techniques to characterise the bacterial and fungal communities associated with the nudibranch *Pteraeolidia semperi* in Singapore. Characterisation of microbial members in the marine environment paves the way for further understanding of how marine microbiomes are structured and the benefits their hosts derive from their microbial communities. Additionally, we identified a module of bacterial families that are likely working together synergistically, allowing *P. semperi* to digest algal food sources more efficiently. Crucially, understanding hosts and their associated microbial communities allows further development and testing of explicit hypotheses to facilitate a better understanding of how species and their associated microorganisms interact and evolve as environments change.

## Data Availability

All samples associated with this work have been deposited in the appropriate repositories with the following NCBI accession numbers: 16S; PRJNA901644, Fungal ITS; PRJNA901647, COI Sanger sequences between ON554204-ON554251.

## Supplementary Materials

The following supporting information can be downloaded at: https://www.mdpi.com/article/10.3390/life12121988/s1, Figure S1: Rarefaction curve for (A) bacteria and (B) fungi reads indicating sufficient sequencing depth to recover ASV diversity in each sample; Figure S2: Stacked bar plot of the top 20 (A) bacterial and (B) fungal phyla for each island sampled; Figure S3: Stacked bar plot of the top 20 (A) bacterial and (B) fungal classes for each island sampled; Figure S4: Stacked bar plot of the top 20 (A) bacterial and (B) fungal orders for each island sampled; Figure S5: Stacked bar plot of the top 20 (A) bacterial and (B) fungal families for each island sampled; Table S1: Total number of sequences in each sample, and the number of sequences after filtering and removing chimeric sequences for bacterial (16S) and fungal (ITS) data; Table S2: ANOVA was conducted on the effect of site on the bacterial (16S) and fungal (ITS) community alpha diversity metrics, Shannon diversity index and species richness, associated with *Pteraeolidia semperi*; Table S3: PERMANOVA conducted on the effect of sampling site on the bacterial (16S) and fungal (ITS) community compositions associated with *Pteraeolidia semperi*, with both Weighted UniFrac and Bray-Curtis distances; Table S4: Multi-level pairwise analysis with Bonferroni correction post-PERMANOVA on bacterial (16S) and fungal (ITS) compositions with both Weighted Unifrac and Bray-Curtis Distances; Table S5: (A) Identities of 16S and ITS ASVs unique to each site and (B) shared across the four sites; Table S6: (A) Network centrality metrics and the identity of the nodes, (B) Weights of the correlation between nodes in the network.
